# Epidemiological manifestations and impact of healthcare-associated infections in Libyan national hospitals

**DOI:** 10.1186/s13756-023-01328-7

**Published:** 2023-11-06

**Authors:** Mohamed Ali Daw, Mahamat Hassabarassoul Mahamat, Said Emhamed Wareg, Abdallah H El-Bouzedi, Mohamed Omar Ahmed

**Affiliations:** 1 Department of Medical Microbiology & Immunology, Faculty of Medicine, University of Tripoli, Tripoli, 82668 CC Libya; 2 Clinical Microbiology & Epidemiology, Acting Physician of Internal Medicine, Scientific Coordinator of Libyan Society of Hospital Infection, Tripoli, Libya; 3 Department of Biological Sciences, Libyan Academy of Science, Tripoli, Libya; 4 Department of Biology, University of Nalout, Nalout, Libya; 5 Department of Statistics, Faculty of Science, Tripoli University, Tripoli, 82668 CC Libya; 6 Department of Microbiology & Parasitology, Faculty of Veterinary Medicine, University of Tripoli, Tripoli, 82668 CC Libya

**Keywords:** Hospital-acquired Infection, Epidemiology, Libyan hospitals, Tripoli Medical Center, Tripoli Central Hospital, Benghazi Medical Center, Sabha Medical Center

## Abstract

**Background:**

Healthcare-associated infection is a serious global problem, particularly in developing countries. In North African countries, comprehensive research on the incidence and effects of such infections is rare. This study evaluated the epidemiology and determined the impact of healthcare-associated infections in Libyan national teaching hospitals.

**Methods:**

A prospective longitudinal study was carried out in Libya’s four largest teaching and referral hospitals (Tripoli Medical Center, Tripoli-Central Hospital, Benghazi Medical Center, and Sabha Medical Center) from November 1, 2021, to October 31, 2022. The epidemiological events and the parameters incorporated in this study were based on the data published by the Libyan Centers for Disease Control. The surveillance was carried out on all patients admitted to the wards of medicine, surgery, intensive care, gynecology & obstetrics, and pediatrics in all four hospitals. Trained staff reviewed the medical records and compared the percentages of patients with healthcare-associated infections. Bio-statistical and multivariable logistic regression analyses were carried out to test the variables associated with healthcare-associated infections and the resulting deaths.

**Results:**

A total of 22,170 hospitalized patients in four hospitals were included in the study. Hospital-acquired infection was reported in 3037 patients (13.7%; 95% CI: 12.9–14.4%). The highest incidence was in Benghazi Medical Center (17.9%; 95% CI: 16.9–18.7%), followed by Sabha Medical Center (14.8%; 95% CI:14.9-16.51%). Surgical site infection was the most prevalent (31.3%), followed by ventilator-associated pneumonia (29.3%), urinary tract infection (26.8%), and bloodstream infection (12.6%). Patients with healthcare-associated infections experienced severe morbidity requiring intervention. New antimicrobial regimens were needed for 1836 patients (93%), and 752 patients (34%) required admission to intensive care. Surgical intervention, respiratory support, and inotropes were also needed as a consequence of HAI events.

**Conclusions:**

The high incidence of healthcare-associated infections in Libyan hospitals should be considered a major problem and a serious burden. This should alert healthcare authorities at the national and hospital levels to the urgent need for preventive and control strategies to combat hospital-acquired infections.

**Supplementary Information:**

The online version contains supplementary material available at 10.1186/s13756-023-01328-7.

## Background

Hospital-acquired or healthcare-associated infection (HAI), formerly called nosocomial infection) has been defined as “an infection occurring in a patient in a hospital or other healthcare facility in whom the infection was not present or incubating on admission to that hospital/facility” [1]. However, depending on the incubation period and the length of stay, some of these infections may be manifested only after patient discharge. The commonly reported HAIs are surgical site infections (SSIs), urinary tract infections (UTIs), pneumonia, and bloodstream infections [2]. A recent European study revealed that of every 20 patients hospitalized, at least one acquired a preventable HAI [3]. Most HAIs involve *Staphylococcus aureus*, *P. aeruginosa*, *Klebsiella pneumonia*, or the *Acinetobacter* species highly resistant to multiple antimicrobials, and the lack of new antimicrobials increases the huge burden in Europe [3, 4]. Similar findings have been reported in Southeast Asian countries, where the overall incidence ratio of HAI was 9.1% and the most common microorganisms were *P. aeruginosa*, *Klebsiella* species, and *Acinetobacter baumannii* [4].

Hospital-associated Infections have a large impact on morbidity and mortality worldwide. They prolong hospital stays, substantially increase healthcare costs, and pose a threat to the safety of patients and healthcare workers [5]. Given the wide range of microorganisms and hospitals involved, reliable data on HAI at national and international levels are difficult to get [5, 6]. Different studies have revealed a fragmented picture of the endemic burden of HAI in the world. Assessment of the global burden of HAI has been hampered by the lack of accurate data describing endemic infections at national and regional levels, particularly in resource-limited countries [6]. An epidemiological study carried out by the World Health Organization (WHO) in 14 countries reported that the overall incidence of HAIs was 7.6% in North America and Europe and 10.1% in Asia, Latin America, and Sub-Saharan Africa [7].

Europe’s overall HAI incidence ratio ranged from 3·5% to 14·8%. About 2–3 million people are affected annually, with an economic burden of €800 million [8]. The Centers for Disease Control and Prevention (CDC) estimated that HAIs in American hospitals account for approximately 1.7 million infections and 99,000 associated deaths each year, with an estimated excess healthcare cost of $28–33 billion each year [9].

In African countries, the magnitude of HAI is not clear and it is complicated by economic constraints and internal conflicts that make it difficult to control [10, 11]. The limited number of healthcare professionals and overcrowding in hospitals result in inadequate infection control practices. On top of these, the lack of infection control policies, guidelines, and trained personnel makes the problem even worse. However, the magnitude of the problem remains largely unknown, and in most cases, it is underestimated due to the complex nature of its diagnosis and the lack of proper surveillance [12].

Only a few African countries have established national surveillance systems for HAI, as emphasized by the WHO patient safety module [13]. In Ethiopia, HAI incidence reached up to 40%. The surgical site, urinary tract, and bloodstream infections were the commonest. The type of surgery, patients’ underlying medical conditions, and the type of ward were important factors associated with increased risk of HAI in Ethiopia [13, 14]. Similar results were reported in West Africa and Tanzania, where the infection incidence reached over 15%, including highly resistant Gram-positive and Gram-negative bacteria [15, 16]. In Uganda, the incidence of HAIs was reported to be over 14%, with UTI and SSI ratios reaching 38% and 21.9%, respectively [17]. However, encouraging results were reported in South Africa, where the overall HAI incidence was only 7.7% [18].

Literature on HAIs in North African countries is scarce. Libya is the second largest country in North Africa and has the longest coast in the Mediterranean basin facing Europe. Studies on HAIs have rarely been reported, and the country has no system for estimating the burden of such infections. The extreme scarcity of data on hospitalized patients in Libya limits the estimation of the impact of HAIs on mortality and healthcare costs. Therefore, this prospective active surveillance study aimed to evaluate the epidemiology and investigate the spectrum, risk factors, and impact of HAI in Libyan referral hospitals. The findings provide baseline information for healthcare providers to implement proper prevention strategies to control the incidence and complications of HAIs.

## Methods

### Healthcare settings

This study was conducted at four teaching and referral hospitals in Libya: Tripoli Medical Center (TMC), Tripoli Central Hospital (TCH), Benghazi Medical Center (BMC), and Sabha Medical Center (SMC). TMC is the largest hospital in the country, with over 1450 beds, and THC has about 1000 beds. Together, these two hospitals serve a population of over four million people. BMC is the largest hospital in the eastern region, with 1200 beds serving over two million people. SMC, located in the southern region, has 1000 beds and serves over one million people.

### Study design and data collection

This is a prospective surveillance study conducted from November 1, 2021, to October 31, 2022. The surveillance of HAI events and data collection followed the surveillance definition of HAI published by the Centers for Disease Control/National Healthcare Safety Network (CDC/NHSN) [19].

The study included all patients admitted to the medicine, surgery, intensive care, gynecology & obstetrics, and pediatrics wards during the study period. Epidemiological information, including demographics (age, sex, hospitalization period), treatment received, and clinical, diagnostic, and HAI event data, were collected from each patient. Data were collected weekly for all patients admitted for 48 h or longer without evidence of bacterial infection at admission. HAI definitions were based on the CDC/NHSN criteria as in our previous studies and only the first infection per patient was counted [10, 20]. They included surgical site infection (SSI), bloodstream infection (BSI), urinary tract infection (UTI), and ventilator-associated pneumonia (VAP) [20]. Trained physicians and senior nurses collected the data under the supervision of a senior clinical epidemiologist from the Department of Medical Microbiology, Faculty of Medicine, Tripoli (M.A. Daw).

### Statistical analysis

Statistical analysis and data management were carried out using Statistical Software version 13.0 IC (Stata Corp LP; College Station, USA). HAI incidence was calculated for a 95% confidence interval (95% CI). Chi-square test and logistic regression analysis were used to test variables for association with HAI events and death from HAI. The statistical level was considered to be significant at a *p*-value of < 0.01.

## Results

A total of 22,170 patients were enrolled from four teaching hospitals in Libya. The distribution of the patients across the hospitals was as follows: 7650 (34.5%) were from TMC, 3921 (17.7%) were from TCH, 6259 (28.2%) were from BMC, and 4340 (19.6%) were from SMC. The clinical and demographic characteristics of the patients are summarized in Table 1. The median age of the patients was 39.6 years, with 11,959 (53.9%) females and 10,211 (46.1%) males. More than 60% of the patients were over 40 years of age, while only 18% were below 30 years. The distribution of the patients across the different wards was as follows: 33.2% in medical wards, 30.3% in surgical wards, 17.3% in gynecology & obstetrics, 12.9% in pediatrics, and 5.7% in intensive care units (ICU). Underlying non-communicable diseases among the patients with HAI were reported in 4368 patients (19.7%), with the following distribution: hypertension (27.3%), cardiovascular diseases (23.9%), diabetes mellitus (22.3%), renal failure (19.7%), and cancer (6.8%).

**Table 1:** Clinical and demographic characteristics of the Libyan patients included in the study

Patient characteristics	Tripoli Medical Center (TMC)	Tripoli Central Hospital (TCH)	Benghazi Medical Center (BMC)	Sabha Medical Center (SMC)	Total (%)
**Patients (n, %)**	7650 (34.5)	3921 (17.7)	6259 (28.2)	4340 (19.6)	22,170 (100.0)
**Sex**					
Male	3380	1951	2850	2030	10,211(46.1)
Female	4270	1970	3409	2310	11,959 (53.9)
**Age (years)**					
< 18	1210	340	723	582	2855 (12.9)
18–30	371	110	360	289	1130 (5.1)
31–40	736	435	613	589	2373(10.7)
41–50	2149	643	1620	1431	5843 (26.4)
51–60	2559	692	2179	1520	6950 (26.4)
> 60	1320	314	875	510	3019 (13.6)
**Ward**					
Medical	3291	897	1789	1379	7356 (33.2)
Surgery	2310	870	1969	1564	6713 (30.3)
OB/Gyn	1870	0.0	1265	841	3976 (17.9)
Pediatrics	1350	0.0	823	682	2855 (12.9)
ICU	521	208	331	210	1270 (5.7)

TMC: Tripoli Medical Center; TCH: Tripoli Central Hospital; BMC: Benghazi Medical Center; SMC: Sabha Medical Center, Ob/Gyn: obstetrics & gynecology

Hospital-acquired infections within Libyan national hospitals (Table 2) were observed in 3037 patients, giving an overall incidence of 13.7% (95% CI: 12.9-14.4%) (Table 2). The highest HAI incidence was observed in BMC (17.9%; 95% CI: 16.9-18.7%), followed by SMC (14.8%; 95% CI: 14.5.9-16.5%) and TCH (14.2%; 95% CI: 13.7-15.8%). The lowest incidence was in TMC (9.4%; 95% CI: 8.7-10.7%). Overall, the intensive care units had the highest HAI incidence (29.8%), followed by the pediatrics and surgery wards (14.7% and 13.7%, respectively). The lowest incidence was found in the medical wards (11.8%) and obstetrics & gynecology wards (11.3%). Table 3 shows the logistic regression analysis to obtain the odds ratio in the presence of more than one explanatory variable. Patients with HIS were more likely to be females, particularly from SMC and BMC, and aged over sixty (OR 0.7201; 95% CI, 0.6350–0.8166).

**Table 2:** Incidence of healthcare-associated infection in patients admitted to Libyan national hospitals

Characteristics	Patients with HAI	Patients without HAI	Total	% HAI	OR	95% CI	Z statistic		*P* value
**Hospital**									
TMC	719	6931	7650	9.4%	0.5461	0.4997 to 0.5967	13.337	P < 0.0001	
TCH	556	3365	3921	14.2%	1.0501	0.9509 to 1.1596	0.966	P = 0.3340	
BMC	1120	5139	6259	17.9%	1.5910	1.4681 to 1.7241	11.327	P < 0.0001	
SMC	642	3698	4340	14.8%	1.1188	1.0182 to 1.2294	2.336	P = 0.0195	
**Ward**									
Medical	871	6485	7356	11.8%	0.7764	0.7138–0.8445	5.903	P < 0.0001	
Surgery	920	5793	6713	13.7%	1.0007	0.9207–1.0877	0.017	P = 0.9862	
Ob/Gyn	448	3528	3976	11.3%	0.7654	0.6879–0.8516	4.910	P < 0.0001	
Pediatric	419	2436	2855	14.7%	1.0970	0.9812–1.2265	1.627	P = 0.1038	
ICU	379	891	1270	29.8%	2.9195	2.5715–3.3145	16.548	P < 0.0001	
**Sex**									
Male	1248	8963	10,211	12.2%	0.7915	0.7324–0.8555	5.900	P < 0.0001	
Female	1789	10,170	11,959	15.0%	1.2634	1.1690–1.3654	5.900	P < 0.0001	
**Age (years)**									
< 18	303	2552	2855	10.6%	0.7201	0.6350–08166	5.117	P < 0.0001	
18–30	36	1094	1130	3.2%	0.1978	0.1416–0.2763	9.503	P < 0.0001	
31–40	83	2290	2373	3.5%	0.2067	0.1654–0.2581	13.891	P < 0.0001	
41–50	774	5069	5843	13.2%	0.9489	0.8693–1.0359	1.171	P = 0.2416	
51–60	1566	5384	6950	22.5%	2.7191	2.5155–2.9392	25.190	P < 0.0001	
> 60	275	2744	3019	9.1%	0.5947	0.5220–0.6775	7.814	P < 0.0001	

TMC: Tripoli Medical Center; TCH: Tripoli Central Hospital; BMC: Benghazi Medical Center; SMC: Sabha Medical Center; HAI: hospital-associated infection; OR: odds ratio; Ob/Gyn: obstetrics and gynecology

**Table 3:** Logistic Statistical Analysis of the variable factors associated with Hospital-acquired Infection in Libyan national hospitals

Statement effect factor	probability ratio Exp(B)	Significance Level P-value	regression coefficient (B)	OR	Confidence interval 95%
**Hospitals**					
TMC	0.435	P < 0.0001	-0.021	0.5461	0.4997 to 0.5967
TCH	0.851	P = 0.3340	0.001	1.0501	0.9509 to 1.1596
BMC	1.531	P < 0.0001	-1.107	1.5910	1.4681 to 1.7241
SMC	1.0176	P = 0.0195	-0.087	1.1188	1.0182 to 1.2294
**Ward of admission**					
Medical	0.519	P < 0.0001	-1.072	0.7764	0.7138–0.8445
Surgery	0.913	P = 0.9862	1.091	1.0007	0.9207–1.0877
Obstetrics& gynecology	0.718	P < 0.0001	-001	0.7654	0.6879–0.8516
Pediatric	0.897	P = 0.1038	0.194	1.0970	0.9812–1.2265
ICU	1.783	P < 0.0001	-0.001	2.9195	2.5715–3.3145
**Age group**					
Under 18 year	0.781	P < 0.0001	-0.001	0.7201	0.6350–0.8166
18–30 year	0.139	P < 0.0001	1.092	0.1978	0.1416–0.2763
31–40 year	0.186	P < 0.0001	0.871	0.2067	0.1654–0.2581
41–50 year	0.915	P = 0.2416	0.001	0.9489	0.8693–1.0359
51–60 year	1.958	P < 0.0001	-0.001	2.7191	2.5155–2.9392
Above 60 year	0.372	P < 0.0001	-0.002	0.5947	0.5220–0.6775

**Fig. 1:**
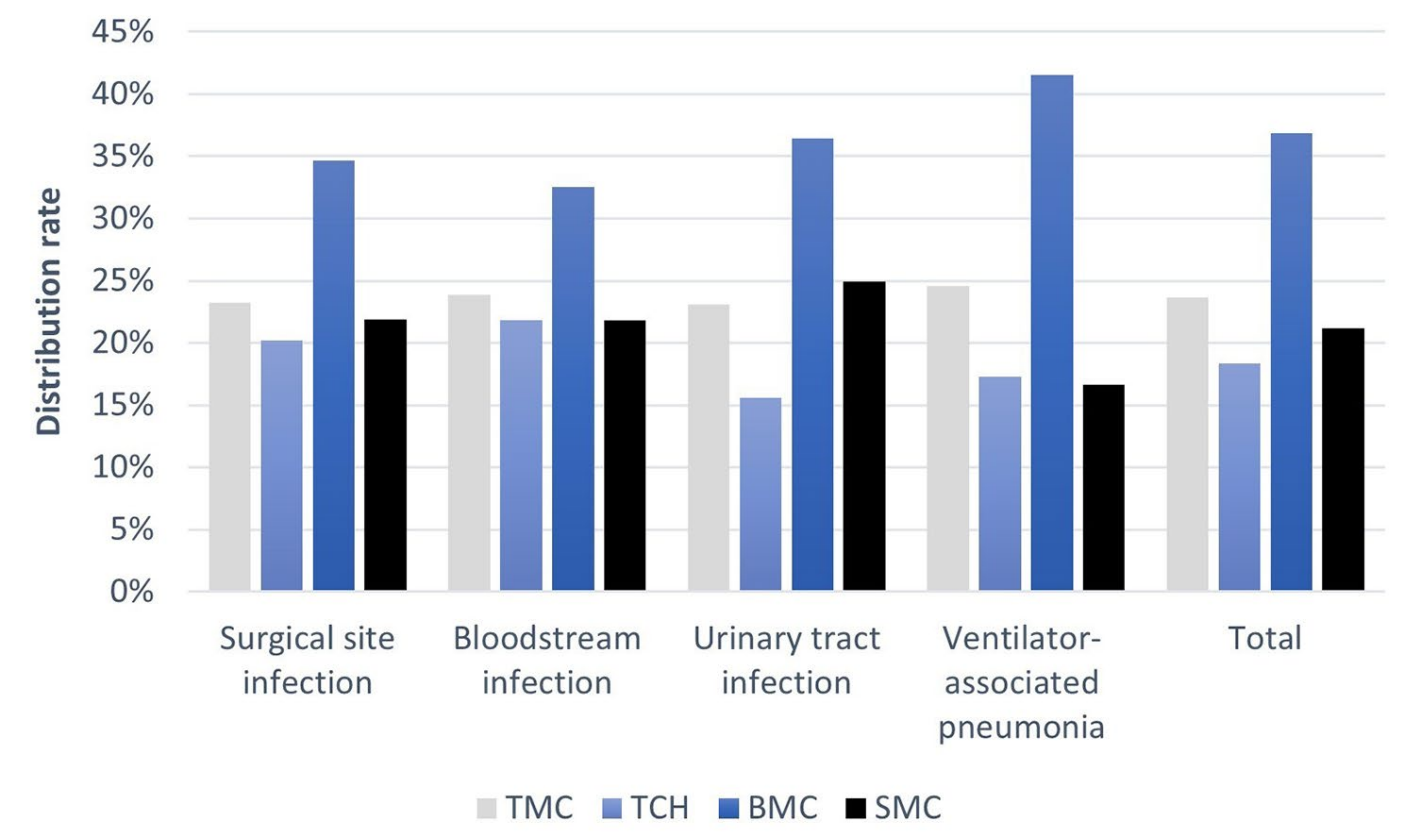
The distribution of health care associated infection sites in each hospital. TMC: Tripoli Medical Center; TCH: Tripoli Central Hospital; BMC: Benghazi Medical Center; SMC: Sabha Medical Center

The incidence of the different types of infections in each hospital is shown in Fig. 1. Ventilator-associated pneumonia (41.3%), and urinary tract infection (37.2%), were the most frequent, followed by surgical site infection (34,7%) and bloodstream infection (22.1%). Benghazi Medical Center reported the highest incidence of all HAI, particularly ventilator-associated pneumonia, followed by Tripoli Central Hospital and then Sabha Medical Center (Fig. 1-Source file 1).

The duration of hospitalization ranged from 3 to 37 days. The mean duration of hospital stay for patients with HAI (14.8 days; SD ± 9.7) was longer than for those without HAI (8.3 days; ± 6.98). There was a significant mean difference in hospital stay of 6.34 days (95% C.I: 5.86–17.41) between these two patient groups.

Different microorganisms were isolated and identified from the HAI patients (Table 4). *E. coli* (24.2%) was the most frequent one, followed by *S. aureus* (20.4%), *Pseudomonas aeruginosa* (11.9%), *Klebsiella* spp. (10.9%), *Enterobacter* spp. (16.4%) and *Acinetobacter* spp. (16.2%).

**Table 4:** Microbiological profile of Hospital Acquired-infection sites among Libyan National Hospitals ,

	*S. aureus*		*E. coli*	*P. aeruginosa*	*Klebsiella* spp.	*Enterobacter* spp.	*Acintobacte*r spp.	Other pathogens	Total no
		**Surgical site infection**							
n (%)	297 (42.6)		79 (11.3)	67 (9.6)	56 (8.0)	158 (22.7)	40 (5.7)	9(1.2)	706
		**Ventilator-associated pneumonia**							
n (%)	78 (12.4)		61 (9.7)	79 (12.5)	49 (7.8)	132 (20.9)	232 (36.8)	7(1.1)	638
		**Urinary tract infection**							
n (%)	29 (7.0)		217 (52.7)	46 (11.2)	49 (11.9)	39(9.5)	32(7.8)	8(1.9)	420
		**Bloodstream infection**							
n (%)	17 (6.7)		82 (32.3)	29 (11.4)	39 (15.4)	28 (11.0)	59 (23.2)	7(2.7)	261
		**Other infections**							
n (%)	94 (17.7)		172 (32.4)	79 (14.9)	83 (15.6)	57 (10.7)	46 (8.7)	11(2)	542
		**Total**							
n (%)	515 (20.4)		611 (24.2)	300 (11.9)	276 (10.9)	414 (16.4)	409 16.2)	42 (1,6)	2567 (100.0)

Some patients with HAI experienced severe morbidity that required intervention (Table 5). New antimicrobial regimens were needed for 1836 patients (93%) and 752 (34%) required ICU admission. In addition to surgical intervention, respiratory support, and inotropes were used when needed.

**Table 5:** Interveinal management of hospital-acquired infection cases in the Libyan national hospitals

Management type	TMC (n %)	TCH (n %)	BMC (n %)	SMC (n %)	Total (n %)
New antimicrobials	421 (89)	327 (92)	671 (96)	417(94)	1836 (93)
Admission to ICU	190 (29)	178 (35)	197 (32)	187(39)	752 (34)
Surgery	153 (14)	97 (17)	139 (13)	107(9)	496 (13)
Inotropic agents	187 (23)	139 (21)	194 (27)	156 (16)	676 (22)
Respiratory support	91 (17)	71 (11)	127 (13)	109 (10)	393 (13)
Device removal	76 (21)	63 (17)	81 (19)	75 (27)	295 (21)

TMC: Tripoli Medical Center; TCH: Tripoli Central Hospital; BMC: Benghazi Medical Center; SMC: Sabha Medical Center

Table 6 shows the mortality associated with healthcare-associated infections in Libyan national hospitals. Overall, there were 386 deaths (12.8%) in patients with HAI, and the highest fatality ratio was reported among patients with ventilator-associated pneumonia (14.8%) followed by patients with bloodstream infection (10.3%), surgical site infection (3.7%) and urinary tract infection (3.1%).

**Table 6:** Mortality associated with healthcare-associated infections in Libyan national hospitals

Type of HA infection	No. of hospital-associated infections	No. deaths	Case fatality ratio
Surgical site infection	751	28	3.7
Ventilator-associated pneumonia	691	102	14.8
Urinary tract infection	512	16	3.1
Bloodstream infection	273	28	10.3
Other	810	19	2.3
**TOTAL**	**3037**	**386**	**12.8**

## Discussion

Herein, we present the first comprehensive study on the incidence and impact of HAI in the four largest referral hospitals in Libya. The overall HAI incidence in the four hospitals was 13.7%. BMC had the highest ratio (17.9%), followed by SMC (14.8%) and TCH (14.2%). The lowest was observed in TMC (9.4%).

HAI incidence varies greatly among countries, in developed countries such as Ireland and Germany reporting a ratio of about 3.5% according to a Euro-surveillance report, while developing countries and Sub-Saharan countries had ratios as high as 21.7% [21–23]. Our data are in concordance with other data reported in North African countries, such as Tunisia, Morocco, and Algeria, as they have an HAI ratio as high as 18%. Therefore, efforts at national and regional levels should be combined to reduce the impact of HAI in these countries [24, 25].

The HAI in Libyan hospitals was found to be 3.1%. It was influenced by sex, age, and ward of admission. Female patients had a higher incidence of HAI, with a 1.25:1 female-to-male ratio, which is supported by other studies conducted in other African countries. Older hospitalized patients (51 years and above) experienced a higher impact of HAI than younger patients, which agrees with studies reported from Russia and Ethiopia [26, 27]. Gram-negative bacilli, particularly *E. coli, P. aeruginosa*, and *Klebsiella* spp., and Gram-positive cocci, mainly *S. aureus*, were the most commonly isolated HAI-causing pathogens, which is consistent with studies from other African countries [22, 23].

Among the different hospital wards, the highest incidence of HAI was observed in the ICU (29.8%) followed by the pediatrics (14.7%), surgery (13.7%), medical (11.8%), and obstetrics & gynecology wards (11.3%). These ratios are similar to those reported from Tunisia and Ethiopia but lower than those in Morocco [28, 29].

The most common type of HAI in our study was ventilator-associated pneumonia (41.3%), followed by urinary tract infection (37.2%) and surgical site infection (34,7%). Those ratios are higher than those reported in the USA and France, but lower than in Nigerian studies [30–34]. The high SSI ratio in our study could be due to various factors, such as ineffective sterilization, inadequate wound dressing, and inadequate procedures during surgery. The admission ratio of patients whose surgery was classified as contaminated was high, particularly at BMC and SMC. The lack or insufficient implementation of guidelines and antimicrobial stewardship in Libya may also contribute to the higher incidence of SSI. Further research is needed to investigate the association between HAI and specific surgical procedures, as well as the implementation of guidelines for antimicrobial use [35].

The pneumonia infection ratio was similar to or lower than in studies carried out in other countries in the MENA region, such as Iran and Saudi Arabia, where pneumonia accounted for 28.9% and 70% of HAI, respectively. It is worth noting that studies in these countries mainly focused on ICU patients, which could contribute to the discrepancy [36, 37]. In our study, the ratio of admission to the intensive care unit varied among the four hospitals. BMC reported the highest ratio (27%), followed by TMC (19%) and SMC (15%).

Urinary tract infection was observed in 37.2%, of the patients. This is a higher percentage than reported in studies conducted in Lithuania (28.5%), Tanzania (31.1%), and Ethiopia (19.8%). Improper catheterization practices may contribute to a high UTI ratio. Several studies have indicated that 79.3% of UTIs can be prevented by avoiding catheterization in hospitals [38–40]. Bloodstream infection was the least frequently reported HAI (22.1.6%), which is in agreement with other studies carried out in North and Sub-Saharan African countries. Inadequate aseptic techniques during the collection of blood specimens or administration of intravenous drugs may contribute to bloodstream HAI. Therefore, proper attention to aseptic techniques is important [5, 41, 42].

Our study revealed that most of the reported HAIs were systemic infections rather than localized wound or soft tissue infection. The overlapping of multiple HAI in patients was also noticeable and thus it deserves specific attention due to the magnitude of the problem and the threat to the patient population getting service at the hospital. In line with this, the most frequently isolated bacteria were *E coli*, *S. aureus*, and *Klebsiella* species. These bacteria are also known to cause community-acquired infection in Libya [20]. This may suggest that such bacteria might have colonized the hospital environment and devices. Therefore, further studies are needed to evaluate the interaction between hospital and community-acquired infections.

HAI has a significant impact on morbidity and mortality. They prolong hospitalization, increase the requirement for ICU admission, and necessitate additional diagnostic and therapeutic interventions. In our study, 34% of the patients were admitted to the ICU, and over 90% needed antimicrobials. These factors led to extended hospital stays, resulting in overcrowding (particularly in the ICU) and difficulties in admitting new patients. Furthermore, patients with bloodstream infections can serve as reservoirs for the transmission of drug-resistant pathogens. Our findings are in concordance with other studies carried out in South African hospitals. These factors reflect the consequences of HAI and may act as predictors of hospital mortality. However, such associations need further study [43–45]. Surgical intervention, respiratory support, and device removal were needed for HAI events. Interestingly, they varied among the hospitals. The highest frequency of inappropriate management of HAI was seen in BMC, followed by SMC and then TMC. This is contrary to studies carried out in developed countries. This could be attributed to a lack of proper management, monitoring, and efficient safety procedures in our study settings [1, 46]. The crude mortality associated with HAI events in our study was high as it reached up to 12.8% compared with other studies published from other developing countries such as South Africa (5,3%) and Indonesia (8%). This may be attributed to a  lack of ICU access, laboratory investigations, and antimicrobials for multi-drug resistant pathogens. This finding is important and deserves further explanation.

### Strengths and limitations

A major strength of this study is its comprehensive perspective of HAI in Libya. To our knowledge, this is the largest study characterizing the epidemiological manifestations of HAI in the MENA region. Our findings should alert the decision-makers to the fact that HAI is endemic and represents a hidden and serious burden for the Libyan healthcare system. Hence, health authorities and decision-makers should take the actions needed to fulfill the requirements of the pledge of WHO’s First Global Patient Safety Challenge [47–49]. On the other hand, several limitations need to be considered when interpreting the results of this study. There could be misreporting of some surveillance data that may not reflect the real situation in the participating hospitals. Another limitation was the inability to follow up with every patient, particularly for surgical site infections, which were highly prevalent. We were unable to investigate the treatment and the clinical outcomes of HAIs due to differences in the hospitals studied [10, 11].

The scope of this study was principally limited by data availability. The incidence of antimicrobial resistance, particularly MRSA-causing HAI is alarmingly high, and resistant infections may be more expensive to treat. However, data limitations prevented us from determining the burden of antimicrobial resistance in the studied hospitals.

Despite these limitations, our study gives good insights into HAI in Libyan hospitals, particularly as the country has been involved in a major internal conflict since 2011. Our findings could be used as a reference for future studies and to plan HAI prevention strategies and research [50, 51]. The problem of HAI in Libya is particularly serious because there are no national programs for HAI prevention or mandatory reporting of data in healthcare settings [11, 20]. Unfortunately, lack of awareness and low levels of staff preparedness and knowledge are the main factors leading to poor infection control in Libya. Furthermore, there is no active surveillance of HAI, which is not seen as a priority in Libyan hospitals, and even in ICU settings. Therefore, education and comprehensive training programs for hospital staff should be provided. National guidelines have to be implemented. Doctors and nurses should be obliged to follow and fulfill the requirements for HAI prevention and control.

## Conclusion and recommendations

The incidence of HAI is high in Libyan hospitals, where pneumonia and surgical site infection were the most common infections, followed by urinary tract infection and bloodstream infection. This is combined with a lack of proper clinical management, resulting in a heavy burden on the Libyan healthcare system. National guidelines for HAI and improvements in microbiological diagnosis and antibiotic susceptibility testing are crucial. The Libyan national health authority should implement appropriate management of HAI. Development of national guidelines for HAI and improvement of microbiological diagnosis and antibiotic susceptibility testing are needed.

### Electronic supplementary material

Below is the link to the electronic supplementary material.

Supplementary Material 1

## Data Availability

All data generated or analyzed during this study are included in this published article.

## Abbreviations

TMC: Tripoli Medical Center
TCH: Tripoli-Central Hospital
BMC: Benghazi Medical Center
SMC: Sabha Medical Center
HAI: Hospital-acquired infection
BSI: bloodstream infection
ICU: intensive care unit
SSI: surgical site infection
UTI: urinary tract infection
CDC: Center for Disease Control
